# Creating Structured Hydrogel Microenvironments for Regulating Stem Cell Differentiation

**DOI:** 10.3390/gels6040047

**Published:** 2020-12-02

**Authors:** David K. Mills, Yangyang Luo, Anusha Elumalai, Savannah Esteve, Sonali Karnik, Shaomian Yao

**Affiliations:** 1 School of Biological Sciences, Louisiana Tech University, Ruston, LA 71270, USA; anumalai77@gmail.com; 2 Center for Biomedical Engineering and Rehabilitation Science, Louisiana Tech University, Ruston, LA 71270, USA; Savannah.esteve@gmail.com; 3 Molecular Sciences and Nanotechnology, Louisiana Tech University, Ruston, LA 71270, USA; yangyang317luo@gmail.com; 4 Department of Mechanical and Energy Engineering, IUPUI, Indianapolis, IN 46202, USA; karnik.sonu01@gmail.com; 5 Comparative Biomedical Sciences, Louisiana State University, Baton Rouge, LA 70803, USA; shaomia@lsu.edu

**Keywords:** biomaterials, biopolymers, differentiation, microenvironments, polyelectrolytes, stem cells, substrates, therapeutics

## Abstract

The development of distinct biomimetic microenvironments for regulating stem cell behavior and bioengineering human tissues and disease models requires a solid understanding of cell–substrate interactions, adhesion, and its role in directing cell behavior, and other physico-chemical cues that drive cell behavior. In the past decade, innovative developments in chemistry, materials science, microfabrication, and associated technologies have given us the ability to manipulate the stem cell microenvironment with greater precision and, further, to monitor effector impacts on stem cells, both spatially and temporally. The influence of biomaterials and the 3D microenvironment’s physical and biochemical properties on mesenchymal stem cell proliferation, differentiation, and matrix production are the focus of this review chapter. Mechanisms and materials, principally hydrogel and hydrogel composites for bone and cartilage repair that create “cell-supportive” and “instructive” biomaterials, are emphasized. We begin by providing an overview of stem cells, their unique properties, and their challenges in regenerative medicine. An overview of current fabrication strategies for creating instructive substrates is then reviewed with a focused discussion of selected fabrication methods with an emphasis on bioprinting as a critical tool in creating novel stem cell-based biomaterials. We conclude with a critical assessment of the current state of the field and offer our view on the promises and potential pitfalls of the approaches discussed.

## 1. Introduction

Musculoskeletal tissue injury is a significant problem for patients throughout the world. In the US alone, more than 21 million patients each year are affected by cartilage injuries [1]. There are very few viable options for patients with damaged articular cartilage and major bone loss, and many of the current treatments are invasive. Additionally, their long-term efficacy remains unclear [2,3]. Similarly, for bone, millions of patients in the US undergo procedures to treat bone injuries, deformities, and defects due to disease each year. Bone tissue is second only to blood as the most commonly transplanted tissue, and autografts have become the “gold standard” for treating these types of osseous defects [4,5]. However, there is limited tissue available for autografts, and significant disadvantages of autografts include donor site morbidity, donor site pain, and increases of blood loss and operative time. The ability to generate new tissue for musculoskeletal repair is a significant clinical need. Although tissue engineering has progressed significantly in recent years, the promise of engineering viable tissues has yet to become a clinical reality [6,7]. Stem cells hold great promise for the treatment of diseases that are untreatable at present, enhance treatment modalities for musculoskeletal injuries, offer new therapeutic targets, and as in vitro (disease) models for drug discovery and trials [8].

**Figure 1:**
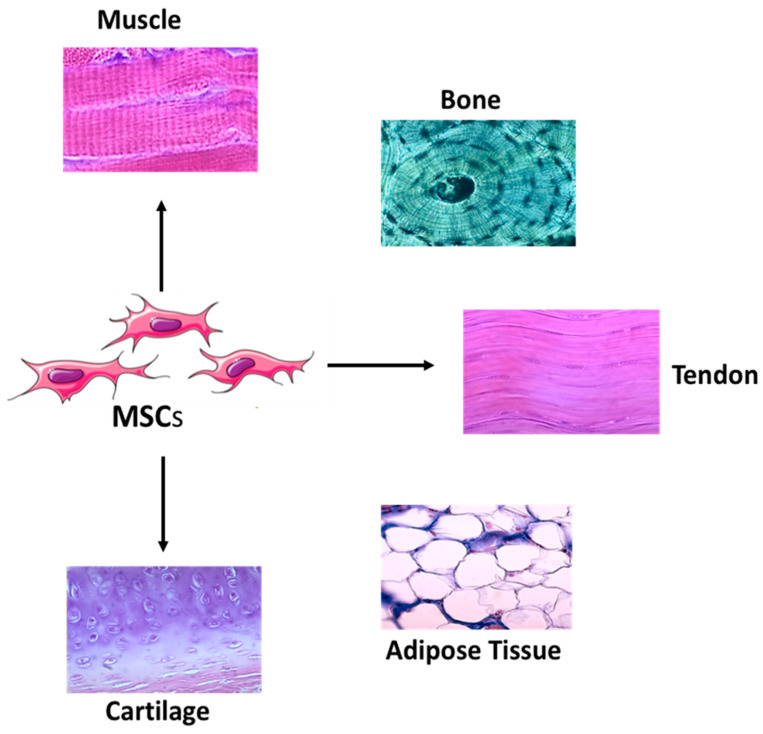
Mesenchymal stem cells, such as bone marrow-derived mesenchymal stem cells and adipose-derived stem cells have the potential to differentiate into various tissue lineages, making them invaluable tools in regenerative medicine and tissue engineering.

Mesenchymal stem cells (MSCs) are the most commonly used cell type in regenerative medicine due to their distinctive capability to self-renew and produce differentiated progeny during development and throughout an organism’s lifespan [9]. MSCs are traditionally known to differentiate into a variety of cell types, including adipocytes (fat cells), chondrocytes (cartilage cells), myocytes (muscle cells), osteoblasts (bone cells), and tenocytes (tendon cells) (Figure 1) [9]. MSCs more recently have been shown capable of evading the immune system as they lack MHC Class II antigens. They can also modulate the immune system and inflammatory cascades, thus enabling the regeneration of tissues and organs to suppress immune-related diseases [6,8,9]. Despite extensive research and our ever-growing knowledge in stem cell biology, the field is still confronted by a lack of reproducible and reliable methods to control stem cell behavior. Although most studies have employed growth factors to induce the differentiation of MSCs, other vehicles influence stem cell differentiation and include cell-cell interactions [10], substrate mechanics [11], biomaterial chemistry [12], surface features [12], and applied physical forces [13].

Perhaps the most significant challenges that the field is currently facing are 1) stem cell in vitro maintenance and expansion [14,15,16], controlling stem cell differentiation into specific cell types that possess in vivo functionality [16,17]; and fabricating multicellular constructs that mimic the in vivo tissue microstructure and organization [18,19]. Developmental biology has contributed significantly to our understanding of the “construction rules,” identifying the morphogenetic directions, both genomic and epigenetic, that lead to tissue and organ formation [20,21]. The field of tissue engineering is applying these insights to engineer neotissues and bioartificial organs with the desired functionalities [22]. Innovative developments in additive manufacturing [23], materials science [24], microfabrication [25], and nano- and microcarriers [20] are permitting the stem cell microenvironment to be manipulated with greater precision and tunability.

## 2. The Promise of Manipulating the Microenvironment for Stem Cell-Based Therapies

The tissue microenvironment directs stem/progenitor cell behavior [23,24]. Differentiation of stem cells into clinically relevant cell types requires a thorough understanding of how the microenvironment controls their fate [24]. Traditional cell culture techniques do not provide the required microenvironment for such cell processes as cell-cell interaction, cell migration and differentiation, and proper tissue formation. It is also an inadequate system to understand human disease processes. The key constituents in designing an instructive stem cell microenvironment are adhesion molecules, extracellular matrix proteins, and complex structures such as the basal lamina or basement membrane, which provide cues for directing stem cell behavior. How these cues can be modified to promote cell death, proliferation, and functionality incorporated into bioengineered constructs remain a critical challenge. Hydrogels, for example, offer significant promise in the field of stem cell tissue engineering [19,24]. Naturally derived hydrogels, such as collagen [25] or hydrogel matrices [20], provide the cellular (adhesion ligands) and biochemical cues (cytokines, growth factors) that can recreate the stem cell niche and assist in the fabrication of tailored microenvironments capable of directing stem cell fate [17,19,26].

## 3. Directing Stem Cell Fate

**Figure 2:**
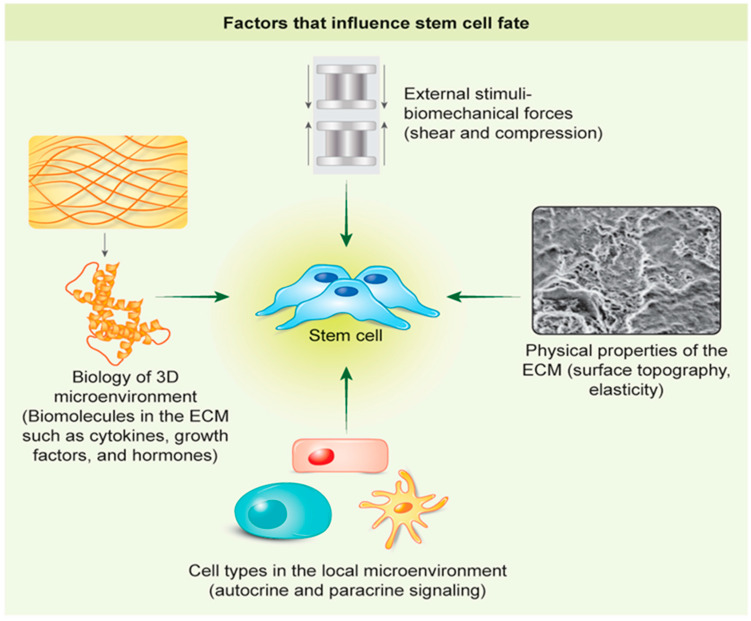
Factors that influence stem cell behavior. There are many influences on stem cells within the local microenvironment that controls or directs stem cell behavior including the extracellular matrix, local signaling agents, mechanical forces, and neighboring cells.

The development of well-defined biomimetic microenvironments for regulating stem cell behavior requires a detailed understanding of the nanoscale properties of polymers, cell-matrix interactions, and the application of environmentally and bioengineering techniques [21,22]. In vivo, cells are exposed to a complex 3D microenvironment containing other cell types, diffusible signals, ECM proteins, the ECM’s biophysical properties, and exogenous stimuli (Figure 2). These are all crucial in directing cell fate, and above all, with an understanding of these influences and cues, we will be able to control or direct stem cell behavior. Tissue regeneration in vitro is far more than selecting a biocompatible biomaterial to support cell growth; additional instructional strategies are usually applied to mimic the native cell microenvironment. For instance, entrapping proteins or peptides on a biomaterial surface to target specific cell receptors. The addition of growth factors enhances cell adhesion, proliferation, and promotes cellular differentiation [27,28]. Creating a novel micro or nanopatterning can also regulate cell morphology and direct cell. behavior [29]. The variations of mechanical properties of biomaterials, such as stiffness and elasticity, also influence cell activities [30,31]. The strategies used for adjusting hydrogel properties are discussed in more detail below.

## 4. Hydrogels-an Overview

**Figure 3:**
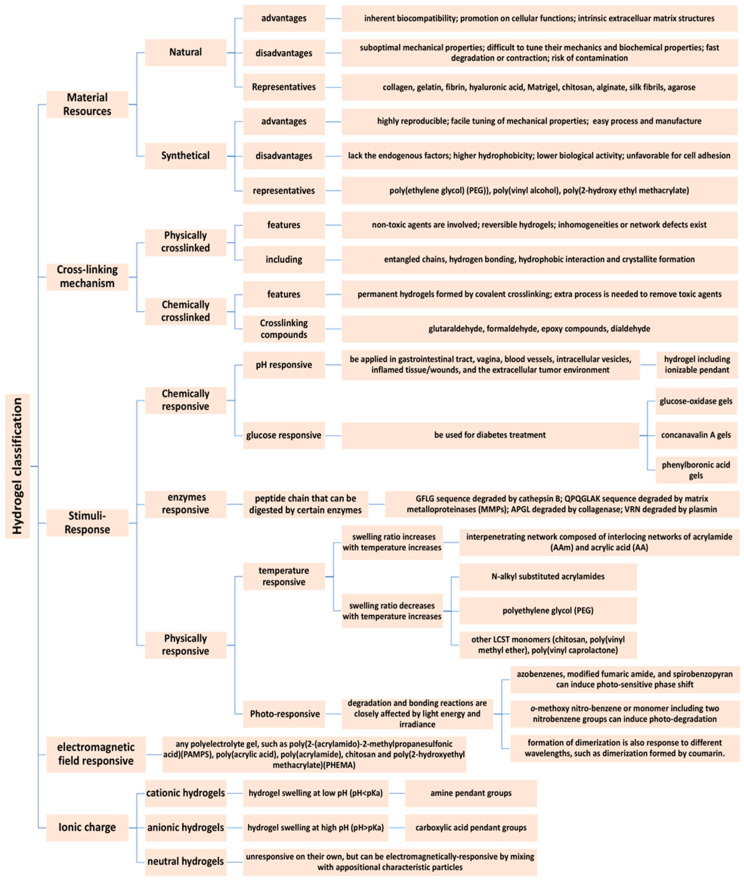
Hydrogel types classified according to chemical and physical properties [36,37,38,39,40].

Several different methods have been developed for creating hydrogels for tissue regeneration and engineering and medical applications. Based on physical or chemical crosslinking, crosslinking, using thermo-sensitive polymers [29], and photochemical reactions have been most commonly used [30,31]. Other methods that have been investigated in hydrogel fabrication include the Michael-type reactions [32], enzyme-mediated reaction [33]. Schiff-base reactions, etc. [22,29]. The compositional and mechanical properties of a hydrogel is critically important in developing a bio engineered tissue (Figure 3) [33,34]. Hydrogel degradation should proceed at a pace that permits new tissue formation and eventual bio-integration into the surrounding tissue without compromising its own physical structure and integrity. Control over hydrogels’ degradation behavior is determined by its polymer composition and can be further moderated through crosslinking, mediated by physical or chemical crosslinking agents [34,35].

Hydrogels used as bioinks for 3D printing of tissue constructs are generally fabricated with lower mechanical strength, making extrusion easier and less impactful on cell viability. However, this prohibits them from being used as an implant in load-bearing microenvironments at a lower mechanical strength. Strategies for designing hydrogels that possess tunable chemistries, novel functionalities, and cell-supportive material properties are discussed below.

## 5. Composite Hydrogels

The material properties of hydrogels can be altered by adding other materials such as clay nanotubes or silver nanoparticles to form a composite hydrogel. Commonly used hydrogels typically have inherently low mechanical strength under loading conditions and have limitations for hard-tissue regeneration because of their low mechanical properties [41,42]. Numerous studies have examined the use of additives in hydrogels to enhance their mechanical properties, deliver a bioactive molecule in situ, or increase their mechanical strength [43,44]. The typical additives are carbon nanotubes [43] or clay nanoparticles [44,45,46] cellulose [47] and metallic nanoparticles [48]. If the composite hydrogels have nanoparticles or nanotubes as part of their composition, they are termed nanocomposite hydrogels [49]. Such composite or nanocomposite hydrogels can be used as biomimetic microenvironment to house MSCs. The composite materials added to these hydrogels can manipulate stem cell behavior in their niches, making them ideal candidates in stem cell therapies [34]. The potential additives used for composite hydrogels are discussed in the following sections.

### 5.1. Nanoparticles

**Figure 4:**
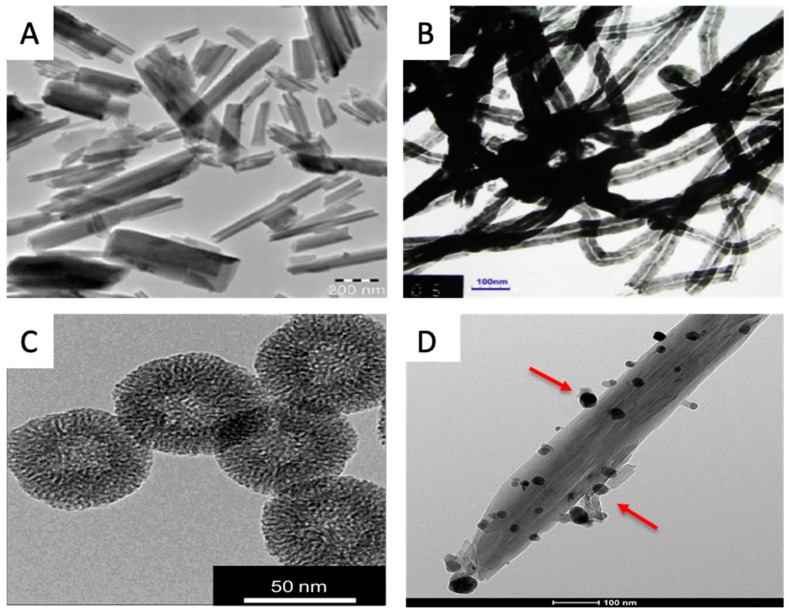
Examples of some representative nanoparticle morphologies. (**A**) halloysite nanotubes; (**B**) carbon nanotubes; (**C**) silica nanoparticles; (**D**) metal nanoparticles. Figures (**B**,**C**) are reprinted with the permission of Elsevier [52,53]. Figures (**A**,**D**) are from the corresponding author’s collections.

Nanotubes and nanoparticles are often used as carriers or vehicles for in situ drug delivery. Their inclusion also improves the material properties of many polymers. They have formed the basis for many tissue engineering applications (Figure 4) [50,51]. The following are common nanotubes and nanoparticles that may be used as additives in hydrogels.

Carbon nanotubes (CNTs) have attracted attention as a potential biomaterial due such unique properties as their high mechanical strength, optical properties, and high electrical conductivity [54]. Many studies have focused on using CNT-based substrates to control stem cell differentiation, and therefore, direct stem cell fate [55]. CNT scaffolds can serve as a support matrix for stem cells’ growth and differentiation, mimicking the native ECM [55,56]. A common approach is to culture stem cells on chemically modified CNTs to direct stem cell behavior [56].

#### 5.1.1. Metallic Nanoparticles

**Figure 5:**
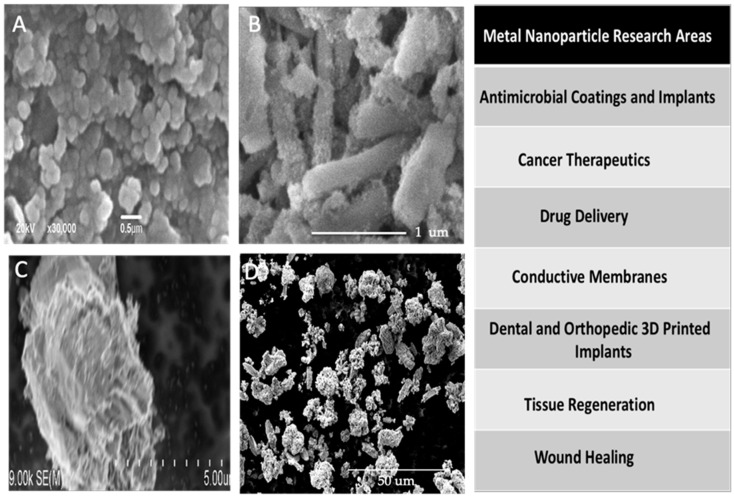
Metal nanoparticle types and areas of research interest. SEM micrographs of some of the more commonly used metal nanoparticles and application areas under intensive research. (**A**) Copper nnaoparticles; (**B**) Iron-coated HNTs). (**C**) Silver nanoparticles; (**D**) Zinc oxide nanoparticles. These nanoparticles have many biological functions and can be applied in multiple applications. Figures (**A**,**D**) are reprinted with the permission of Elsevier [61,62]. Figures (**B**,**C**) are from the corresponding author’s collections.

Metal nanoparticles have been commonly used in the labeling and tracking of stem cells [57] and to trigger stem cell differentiation [57,58]. Magnetic nanoparticles are currently being explored as a means for manipulating stem cell behavior and for 3D building of complex tissues [59]. Gold and silver nanoparticles have seen considerable interest as nanoparticles for a range of biomedical applications in the areas of microbial resistance, anti-cancer, drug delivery, and bioengineering reparative tissues [60] (Figure 5).

##### Iron Oxide Nanoparticles

Iron oxide nanoparticles can be used to facilitate stem cell proliferation, differentiation into different lineages, and migration of stem cells through mechanical stimulation (through mechanotransduction) [63,64]. Sniadecki (2010) coated magnetic nanoparticles with RGD receptors enabling them to bind to receptors on the surface of osteoblasts and applied cyclical magnetic stimulation over a 3-week period in order to deliver nanoscale forces at the ligand-receptor bond [63]. Osteoblasts up-regulated osteopontin, a key bone cell marker, and indicator of osteoblast differentiation, maturation, and matrix mineralization. An interesting study supports the potential of iron oxide MNPs to promote osteogenic differentiation of human bone marrow-derived stem cells using the mitogen-activated protein kinase (MAPK) pathway [65]. The use of a gene microarray assay and bioinformatics analysis revealed that gene expression was widely regulated, and the MAPK signal pathway was activated by IONPs treatment to promote osteogenic differentiation.

##### Silver Nanoparticles

Silver nanoparticles (AgNPs) possess several beneficial features and are widely used as a potent and broad-spectrum inhibitor of anti-microbial activity [66]. AgNPs have been shown to enhance cell proliferation and osteogenic differentiation animal-derived MSCs and promoted fracture healing [67,68]. As an implant coating, AgNPs have also been effective in preventing biofilm formation and promoting bone tissue formation and mineralization on various titanium surfaces [68,69].

##### Gold Nanoparticles

Gold nanoparticles (AuNPs) have relatively low toxicity compared to other NPs. They have attracted much attention recently in biomedicine for use in anti-cancer applications, drug delivery, and for use in stem cell-based regenerative medicine [70]. AuNPs have been shown to regulate MSC differentiation into various cell types, such as osteoblasts [71,72], cardiocytes [72] and neuronal cells [73]. AuNPs have also been used to direct stem cell differentiation. AuNPs act on MSCs to activate the Wnt/β-catenin, ERK/MAPK and p38 MAPK pathways [74,75].

##### Magnesium Nanoparticles

Magnesium (Mg) is a divalent ion found abundantly in the body and plays an important role in many cellular processes [75]. Since it is used in human body as an activator of enzymes, regulation of neuromuscular activities and central nervous system, synthesis of protein, myocardial contraction, and regulation of temperature. Mg is biocompatible and biodegradable and can play a potential role as an implant material [76]. Magnesium alloys has been shown to be an excellent candidate for vascular stents, biodegradable orthopedic implants and hyperthermia [77]. Magnesium nanoparticles has anti-microbial properties and due to the abundance of this metal, it can be cost-effective for clinical use.

When MSCs are cultured in the presence of magnesium, research has observed a decrease in calcium influx and intracellular calcium concentration [75]. Mg has been shown to have a positive effect on cell coverage of H9-OCT4ESCs [76]. Osteoporosis and osteopenia have been associated with a low concentration of Mg. Since Wnt/β-pathways and activation of Notch signaling are related to bone marrow MSCs osteogenesis, Mg can enhance proliferation in these MSCs and herby increase osteogenesis [77]. Integrins play an important role in the activation of intracellular pathway and cell differentiation. Research showed that magnesium improved the attachment of synovial MSCs to osteochondral defects through integrin α3β1 [71]. Increase in concentration of Mg can stimulate gene expression of TRPM7 to promote osteoblasts proliferation [78].

##### Strontium Nanoparticles

Strontium (Sr) nanoparticles have been established as a metal ion that can lead to a significant improvement in the biological and mechanical properties of many polymers [79]. The growing interest of strontium and nanoclays as implants is based on the effects of Sr on cells. It has been shown to induce osteogenic and osteoinductive responses in stem cells. Sr is similar to calcium and hence it is mediated by the calcium sensing receptor (CaSR) expressed in osteoblasts and osteoclasts. Sr can activate intracellular signaling pathways resulting in differentiation and proliferation of MSCs and osteoblasts [80]. It can also lead to an increased mineralization and deposition of extracellular matrix. Sr is now used in dental implants and in orthopedic coatings. Sr may also lead to a heightened bone healing response after further remodeling.

The signaling of vascular endothelial growth factor is essential for stem cell commitment. Research has found that Sr triggers the secretion of this growth factor, which is associated with RhoA/Rac 1 activation, and thereby repressing adipogenesis and activating osteoblastogenesis in a microgravity-induced alteration of cell commitment [81]. Sr treated MSCs were reported to increase phosphorylation of MAPK ERK1/2 and p38 along with osteogenic differentiation. One of the members of the MAPK family is the ERK1/2 (extracellular signal-related kinase) involved in the cellular response to apoptotic promoting signals [82]. Strontium ranelate in lower doses can enhance osteogenic differentiation of human adipose-derived stem cells (hASC) but with higher doses can cause hASC apoptosis by activating the ERK signaling pathway [83]. This information lay a solid foundation for Sr containing scaffolds for use in bone tissue engineering and bone defect repair.

##### Zinc Nanoparticles

Zinc (Zn) has a multitude of physiological functions in the human body. Zinc and alloys containing zinc are progressively promising biomaterials for orthopedic and dental applications [84] including the use of zinc alloys as biomaterials for making scaffolds mimicking mammalian bone. Zinc leads to an increased ECM mineralization in MSCs and there is a concentration-dependent regarding SMCs. Ref. [84,85] Studies have shown that cells preferred zinc ions on the interface of biomaterials rather than it being in diffused state when measured as an expression of zinc transporters (ZnT1 and ZIP1) [84]. Further research on zinc supplementation in osteogenesis in MC3T3-E1 cells showed increased collagen deposition and mineralization [85]. Zinc phosphate has anti-bacterial properties and can assist in preventing bacterial colonization, when loaded on barrier membranes. For osteoblastogenesis, these actions can be regulated by zinc through the TGF-β/Smad signaling pathway [86]. The increased osteogenic differentiation and mineralization effect of Zn makes it an excellent candidate as coatings on implants to promote integration (osseointegration) and prevent bacterial adhesion [87].

Adipose-derived (AD)-MSCs can differentiate into chondrocytes, osteoblasts, and neuron-like cells. Supplementation by Zn can increase AD-MSCs proliferation and neurite outgrowth [87]. ERK1/2, BDNF, and JMK are some of the potential molecules involved in the action and regulation of Zn [88]. Wang et al. (2007) reported that Zn2+ decreased adipocytic cell formation in mouse bone marrow stem cells leading to mineralization, osteoblast proliferation, bone formation, and inhibition of bone resorption [89].

#### 5.1.2. Nanoclay

##### Laponite

Laponite (Na0.7(Mg5.5Li0.3) Si8 O20(OH)4), a human-made nanoclay, has been used in bone tissue engineering. Laponite’s key feature is its high-aspect-ratio nanoplatelet morphology (~25 nm wide and 1–2 nm thick [90]. Their negatively charged surface (due to OH groups) makes them readily dispersible in the water at low concentrations. Laponite was used to reinforce hydrogels for biomedical applications [91,92]. Laponite reinforced nanocomposites have been shown to support human mesenchymal stem cell adhesion and enhanced in vitro mineralization. This feature has seen increased use in bone tissue engineering applications [92].

##### Montmorillonite

Montmorillonite (MMT) is a three-layered smectite group of minerals [93]. MTT nanoparticles are plate-shaped, typically 1 nm in thickness and 0.2–2microns in diameter [93,94]. MTT consists of two tetrahedral sheets covered by one octahedral sheet sandwiched in between. Like many nanoclays, its surface is slightly negatively charged because oxide anions dominate the charge balancing anions present in the interface domain and impart a light overall negative charge to the surfaces of the sheet clay minerals [93,94]. Montmorillonite has an excellent absorption property and available within its interlayer spaces and on the outer surface and edges [93,95]. Montmorillonite has shown promise as an additive in bone tissue engineering applications [93,94,95]. Demir (2016) combined PCL with strontium (Sr)-modified MMT to fabricate a composite scaffold for bone tissue engineering [94]. Chitosan-based clay composites were developed using MTT and hydroxyapatite (HA) as the major constituents [95]. An increase in mechanical strength and a favorable cellular response by osteosarcoma cell line was observed [95]. Chitosan/hydroxyapatite-zinc oxide nanocomposites were also developed by Bhowmick et al. (2017). Their study showed enhanced strength after MTT addition and the composites had an anti-bacterial effect and were cyto-compatible [96].

##### Halloysite

Halloysite nanotubes (HNTs) are a naturally occurring aluminosilicate clay with an external diameter of 50 nm, an inner lumen of 15 nm, and a length of 500–1000 nm [97]. HNTs are commercially available, regarded as GRAS by the FDA, and since 2006 have attracted increasing research interest for use in various applications [97,98]. HNTs have been used in various medical applications, including drug delivery, bioprinting, and tissue repair and regeneration. Primarily due to their cyto- and biocompatibility [99,100]. The HNT lumen enables this nanoparticle to serve as a nanocontainer to load and release a range of biologically active molecules [45,46,97,101]. Halloysite nanotubes have been used in pre-osteoblast-seeded alginate hydrogels to deliver growth factors such as BMP-2, 4 and 6 for up to five days of sustained release at picogram levels [45]. BMP-4 provided a marked stimulus for osteoblast functionality comparable to BMP-6 in terms of osteoblast differentiation and mineralization. However, BMP-4 and 6, in combination, showed a marked enhancement in osteoblast differentiation and functionality [45]. Robinson et al., (2016) developed a nanocomposite consisting of alginate, chitosan, and BMP-2 doped HNTs and showed that osteoblast differentiation was enhanced with BMP-2 release [101]. The authors also proposed that while the focus in this study was on bone regeneration, the design permits local control the behavior of varied cell types and allow the engineering of complex tissues using a single stem cell source. Clay nanotubes have also been used to enrich the calcium phosphate-alginate-chitosan composite hydrogels to deliver anti-microbial agents for extended period of over 24 h making the hydrogel less susceptible to bacterial attachment and biofilm formation [100]. There were also shown to have a key role in tissue engineering [102] and a variety of other biological and medical applications [99].

## 6. Micropatterned Hydrogels

Another class of hydrogels that have been explored to control the stem cell microenvironment is micropatterned hydrogels. Micropatterning is a technique that modifies the homogenous micro-architecture of hydrogels by creating a pattern within the hydrogel, generally with a resolution in microns [103]. Micropatterning can be achieved by using lithography, photomasking, and micromolding [103,104]. Patterning and templating techniques enable precise control over extracellular matrix properties, including composition, mechanics, geometry, cell-cell contact, and diffusion. Depending on the choice of hydrogel polymers used, the micropatterned hydrogels can provide stem cells with a defined microenvironment mimicking the topography and morphology of the native tissue [104,105]. The pattern can provide a good substrate for cell attachment or can embed instructional proteins that promotes stem cell proliferation and differentiation into a mature tissue type [105].

A micropatterned hydrogel made of human tropoelastin was produced to resemble cardiac tissue, which had elastic mechanical support that mimics the dynamic mechanical properties of cardiac muscles [106]. Results demonstrated that cell attachment, spreading, alignment, function, and intercellular communication of cardiomyocytes were enhanced. Patterned hydrogels using dynamic mask microstereolithography and a digital micromirror device were created with dynamic photomasks for crosslinking geometrically specific poly-(ethylene glycol) (PEG) hydrogels [106]. Enhanced cell survival, migration, and neurite growth and guidance were observed. Moreover, the authors describe the method as cheap, quick and easy to use, and can be used with many hydrogels and cell types. Micropatterned PEG-based hydrogels of various compositions have also been produced using various patterning methods (UV embossing, UV photopatterning, and photocuring) [105,106,107]. These have served as the basis for the study of cellular bioactivity in response to micropatterning surfaces. Shah et al., used UV photopatterning of bioactive heparin-based hydrogels formed by UV-initiated thiol–ene reaction between thiolated heparin and diacrylated poly(ethylene) glycol with hepatocyte growth factor premixed into the prepolymer solution. Hepatocyte adhesion and functionality were maintained for over a week [108].

Another method of micropatterning is 3D printing hydrogels using a scanned image of the tissue to be regenerated [109,110]. The pattern, resembling the surface topography of the native tissue, can be scanned, copied on a mask and transferred to a micromold [109]. Tekin et al., 2011 used a dynamic micromolding technique to fabricate sequentially patterned hydrogel microstructures by exploiting the thermoresponsive properties of poly(N-isopropylacrylamide)-based micromolds [110]. The molds can also be used to incorporate chemicals or encapsulate cells into the sequentially patterned hydrogel microstructures [110]. Micromolding is methods that can be used to make hydrogels with the pattern of interest imprinted on them. In many cases, specific tissue patterns could be fabricated as a template for directional cell growth.

## 7. Responsive Hydrogels

The self-assembling peptide hydrogel is another important class of synthetic hydrogels that was first introduced by Zhang et al. (1995) [111]. In this system, polypeptide assemblies form gel-like materials and are composed of short charged oligopeptides that rapidly form insoluble fibers or other kinds of nanoscale structures in the presence of exogenous ions [112,113,114]. These hydrogels provide several unique advantages, such as the ability to form gels and relatively easy gel functionalization, compared to the aforementioned synthetic polymer hydrogels [106]. Examples include three-dimensional scaffolds and nanofibrous networks for tissue engineering comprises ionic self-complementary peptides, which form stable β-sheet structures that self-assemble to form nanofibers [112,113,114]. These nanofibers form interwoven matrices that further form a high-water-content scaffold hydrogel and hold much promise for cartilage tissue engineering.

Peptide hydrogels have been studied for use in cartilage tissue engineering using fully differentiated chondrocytes and MSCs [114,115,116]. As shown in these studies, chondrogenic differentiation was enhanced, followed by extensive cartilage matrix protein synthesis within the peptide hydrogels as compared with control hydrogels. Regeneration of other tissue types has also been demonstrated [117,118,119,120,121]. For example, excised cells were added to Purmatrix™ hydrogel and applied to an injured site where the cells retained normal morphology and function, and multiplied to form new epithelial and subepithelial layers together with the basement membrane [117]. The use of self-assembling peptide hydrogels is now being studied for use in 3D bioprinting applications [113]. For an extensive review, please see Ming and Hauser, (2014) [120].

### 7.1. Thermo-Sensitive Hydrogels

Thermo-sensitive hydrogels have also been examined to control the stem cell microenvironment [121]. Thermo-sensitive hydrogels are liquid at room temperature (23 °C) and form a gel after administration into the body. The temperature change from room to body temperature (37 °C) causes the phase change [121,122]. Different polymers have different critical solution temperatures, the temperature at which the polymer solution undergoes a phase separation [123]. Below the lower critical solution temperature (LCST) polymers are soluble, and above the temperature they become hydrophobic and insoluble, causing the gel formation. Heating above the LCST initiates drug release from the hydrogel, whereas cooling below the LCST collapses the hydrogel, stopping drug release [124]. PCL-g-P(NIPAAm-co-HEMA) micelles were created to carry hydrophobic drugs, such as prednisone acetate, that are typically unstable in physiological environments [124]. With a LCST slightly below body temperature, the micelles gradually released the anti-inflammatory drug over about a 120-h period and temperature-dependent properties allow for gradual drug release and can be used in many other useful applications.

Thermo-sensitive polymers are currently being studied in 3D printing applications [123]. Since 3D printing requires a material that can have diverse mechanical properties and mimic native tissue, thermo-sensitive polymers can fulfill these requirements [125]. Three-dimensional bioprinting is a fabrication method that can create scaffolds similar in structure to native tissues, thus improving the scaffolds functionality by allowing the placement of cells, biomaterials, and bioactive cues on the scaffold [125]. This process is performed with materials called bioinks, which must have special mechanical properties [126] For example, thermoresponsive polymer poly(*N*-isopropylacrylamide) grated hyaluronan (HA-PNIPAAM) with methacrylated hyaluronan (HAMA) creates a thermo-sensitive hydrogel that is liquid at room temperature and gel at body temperature. This property allows for the simple loading of the cartridges of the bioprinter [123]. It was used to create viable 3D printed scaffolds for the stem cell environment [125].

### 7.2. PH-Sensitive Hydrogels

PH-sensitive hydrogels are also being studies as a means for controlling the stem cell environment [123]. These hydrogels are modified to respond to environmental pH changes, causing swelling or collapse depending on the acidity or basicity of the surroundings. Varying the amount of polymer crosslinking changes the hydrogel’s swelling properties, which controls the release of substances [126,127]. For example, pH-sensitive hydrogels are often used for oral delivery of therapeutic peptides and proteins. Specifically, glycopolymers developed by free radical photopolymerization of methacrylic acid and 2 methacryloxyethyl glucoside using tetra (ethylene glycol) dimethacrylate as the crosslinking agent can be used to treat insulin deficiency [128]. Polyanions such as these glycopolymers remain collapsed in acidic environments and then swell in basic or neutral environments. Although the hydrogels are collapsed, they keep the drugs securely inside. When they swell, the drugs are released into the environment. The transition for the glycopolymers occurred at a pH level of 5 [128,129]. Polycationic hydrogels work similarly, except swelling is minimal at neutral pH’s and drug release occurs in acidic environments such as the stomach. This configuration is useful in the delivery of antibiotics. Therefore, pH-sensitive hydrogels can be modified to release different drugs in areas of the body with varying pH levels [130].

## 8. Concluding Remarks

One of the ultimate goals of stem cell research is to use them for tissue regeneration to repair damaged (or diseased) tissues/organs. The ability to control stem cell behavior (proliferation and differentiation) is critical in this regard. A major application of hydrogels is to use them as supportive scaffold materials in tissue engineering and biofabrication. Manipulating hydrogels to create a microenvironment that promotes stem cell proliferation and differentiation in a controlled or regulated fashion is essential. For tissue engineering applications, the hydrogel should be able to the mimic extracellular matrix for stem cells to reside, proliferation and differentiate into desired cell types with proper functionality. Combing 3D bioprinting to fabricate living tissues or organs is an attractive strategy to overcome the donor organ shortage. In addition to building hydrogels that are conducive to stem cell proliferation and differentiation, hydrogels can also be made to act as homing mechanism with loaded chemoattractants designed to recruit stem cells to the site of injury. Development of printable and biocompatible hydrogels that can be used for formulating bioinks for 3D bioprinting is an important future research direction.
